# DVC interneuron cGAL driver in Caenorhabditis elegans

**DOI:** 10.17912/micropub.biology.000082

**Published:** 2019-03-06

**Authors:** Jun Young Oh, Shahla Gharib, Jonathan Liu, Han Wang, Paul Sternberg

**Affiliations:** 1 Division of Biology and Biological Engineering, California Institute of Technology. Pasadena CA 91125

**Figure 1.**
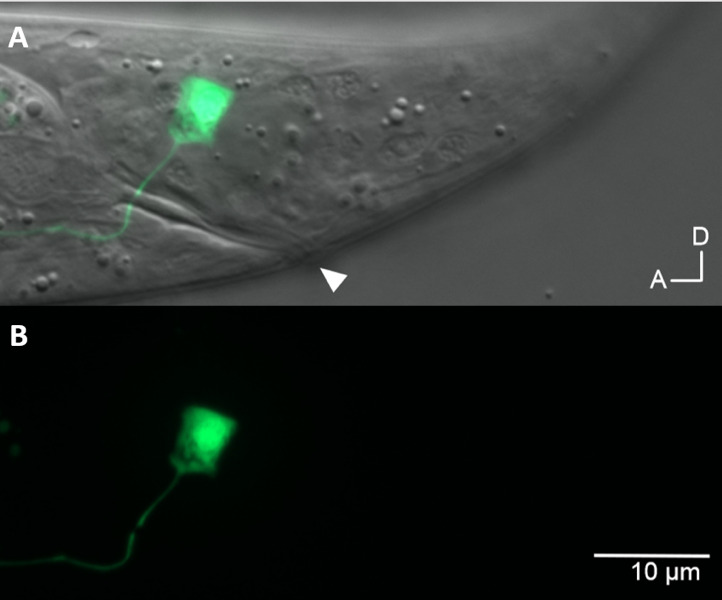
Expression of GFP in the DVC neuron in the tail of a L4 hermaphrodite *Caenorhabditis elegans*. The integrated cGAL driver line (PS8129) using a *ceh-63* promoter was crossed with the UAS-GFP effector strain (PS6843) and double homozygous animals (PS8131) containing both the driver and effector were imaged. GFP, DIC overlay (A) and GFP only (B) images are shown. Arrowhead indicates anus.

## Description

cGAL, a recently developed temperature-robust bipartite GAL4-UAS system in *C. elegans*, consists of two components: a cGAL “driver” that expresses the cGAL protein in specific cells using a promoter (i.e. neuron-specific or tissue-specific), and an “effector” that carries a gene of interest downstream of UAS (Wang *et al*., 2017). Crossing or combining a driver with an effector leads to the expression of the gene of interest in a cell-specific or tissue-specific manner.

Here we report a new cGAL driver for the DVC interneuron. The *ceh-63* promoter was chosen due to its restricted expression in the DVC neuron (Feng *et al*. 2012). The DVC interneuron driver construct containing the *ceh-63* promoter (646 bp upstream of ATG translation start site) was injected into N2 and an integrated DVC driver line was generated. When crossed with the UAS-GFP effector strain (PS6843), the *ceh-63* cGAL driver dictated GFP expression in the single DVC neuron (Figure 1), in addition to GFP in the coelomocyte from P*unc-122::gfp* co-injection marker. We did not observe GFP expression in uterus as reported by Feng *et al.,* 2012.

## Methods

*Molecular cloning*:

pcGAL0073 (P*ceh-63::cGAL*) driver plasmid was constructed from pcGAL0013 (P*rab-3::cGAL*) vector (Wang *et al*., 2017). 646 bp *ceh-64* promoter upstream of isoform a ATG translation start site was obtained through PCR of N2 genomic DNA using NEB Phusion High Fidelity Polymerase with the forward primer 5’ CCCGGCCGGCCGAGACCGAATCAGCACCACC 3’ and the reverse primer 5’ CCCGGCGCGCCGCTAACAACACAATGAGCAAAACAG 3’. FseI and AscI restriction sites were added to the 5’ ends of the primers in order to ligate the *ceh-63* promoter PCR product into the vector. Both the vector and the *ceh-63* promoter PCR product was digested for 45 min at 37°C with FseI and AscI, and ligated using NEB T4 DNA ligase. The *ceh-63* promoter sequence in pcGAL0073 was confirmed by Sanger sequencing (Laragen, CA).

*Injection mix*: 25 ng/μl of pcGAL0073 was mixed with 30 ng/μl of P*unc-122::gfp* co-injection marker and 145 ng/μl of 1kb DNA ladder carrier (NEB, MA). The mixture was injected into fifteen N2 animals and a stable extrachromosomal array line was obtained for integration by X-ray irradiation (Evans 2006), generating *syIs530*.

*Florescence imaging*: DVC neuron was imaged using Zeiss Imager Z2 under a Zeiss Imager Z2 with an Apotome 2.0 and a 100x oil objective using Zen Blue 2.3.

## Reagents

**Table:** 

**Strain**	**Genotype**	**Additional information**
PS8129	*syIs530* II	Outcrossed three times
PS8131	*syIs530* II*; syIs300* V	Slightly sterile, not outcrossed. DVC driver with GFP effector.
